# Navigating Anticoagulation Decisions in Atrial Fibrillation and Frailty Comorbidity

**DOI:** 10.3390/life16091387

**Published:** 2026-08-23

**Authors:** Ziyang Wu, Da Cao, Chengchun Tang

**Affiliations:** 1 Department of Cardiology, Beijing Hospital, Peking Union Medical College, Beijing 100730, China; 2 Department of Emergency, Zhongda Hospital, Southeast University, Nanjing 210009, China; caodacd2018@163.com; 3 Department of Cardiology, Zhongda Hospital, Southeast University, Nanjing 210009, China

**Keywords:** atrial fibrillation, frailty, anticoagulation, direct oral anticoagulants

## Abstract

Frailty is highly prevalent in older patients with atrial fibrillation (AF), complicating anticoagulation decisions due to the dual elevation of both stroke and bleeding risks. This narrative review provides a framework to guide these decisions. We propose a three-step approach: (1) systematically assess frailty using a validated tool to stratify patients into risk categories; (2) translate this stratification into therapeutic choices, generally favoring direct oral anticoagulants (DOACs) over warfarin, with data suggesting a favorable bleeding profile for apixaban in frail populations, but routine switching to a DOAC should be avoided in frail patients who are stable on warfarin with good time in therapeutic range; and (3) consider specific alternatives, such as very-low-dose edoxaban, for patients with severe frailty or a prohibitive bleeding risk, while noting that this strategy should be applied within its specific regulatory and clinical context. The net clinical benefit of anticoagulation, defined as the absolute risk reduction in ischemic stroke offset by the absolute risk increase in major bleeding, diminishes with increasing frailty severity and may vanish in the most severe stages. Clinical decisions must therefore integrate dynamic frailty reassessment, multidisciplinary collaboration, and patient preferences. This review underscores a shift from purely guideline-driven to patient-value-driven individualized anticoagulation strategies in frail older adults with AF.

## 1. Introduction

Atrial fibrillation (AF) is the most common cardiac arrhythmia in the elderly population, with its prevalence increasing markedly with advancing age, exceeding 10% in those aged over 80 years, and a median age at diagnosis of approximately 75 years [1]. Older patients with AF frequently present with coexisting frailty, multimorbidity, and polypharmacy, which not only elevate the risks of stroke and bleeding but also considerably complicate therapeutic decision-making [2].

The prevalence of frailty among individuals with AF has been estimated at approximately 39.7% [3]. Frailty not only significantly heightens physiological vulnerability but also is associated with a 2-fold to 3-fold increase in all-cause mortality, constituting an independent poor prognostic factor that warrants due attention in clinical management. Accordingly, the management of AF in older adults must transcend a purely age-based approach, incorporating frailty assessment into the formulation of individualized anticoagulation strategies.

Patients with AF complicated by frailty face a dual high-risk dilemma, rendering anticoagulation decisions particularly challenging [4]. We performed a search of the PubMed database for English articles published up to June, 2026, using keywords related to ‘atrial fibrillation,’ ‘frailty,’ and ‘anticoagulation.’ We also manually screened the reference lists of relevant articles and selected those we considered most informative and impactful to illustrate the current state of knowledge and clinical challenges. This narrative review summarizes contemporary evidence regarding anticoagulation in older adults with AF and frailty, focusing on the balance between thromboembolic prevention, bleeding risk, anticoagulant selection, treatment transitions, frailty assessment, and individualized decision-making.

## 2. Frailty Assessment as the Foundation

The pooled prevalence of frailty in patients with AF is 39.7% and rises to approximately 75% when pre-frailty is included, which is significantly higher than that in the general elderly population (approximately 63%) [5]. Studies have shown that patients with AF exhibit a higher degree of frailty, significantly higher multidimensional prognostic index (MPI) scores, and a greater number of geriatric syndromes compared to those without AF [6]. There is a sex difference in the association between frailty and the risk of incident AF. Frailty was found to predict new-onset AF in older women but not in men (hazard ratio [HR] = 1.78), a discrepancy that may be attributed to sex-specific differences in inflammatory status and obesity-related pathophysiological mechanisms [7].

A real-world cohort study from Shizuoka Prefecture, Japan, revealed that over 90% of patients with AF initiating oral anticoagulant therapy already presented with varying degrees of frailty, among whom 25.9% had severe frailty [8]. Similarly, a Japanese database-based study suggested that nearly 90% of patients starting anticoagulation exhibited frailty. Approximately 10% of healthy individuals progressed to moderate-to-severe frailty, whereas 12.5% of those with severe frailty showed improvement [9]. Frailty is dynamic, with clinically meaningful transitions between robust, pre-frail, and frail states occurring over time.

The frailty status in patients with AF is associated with adverse prognosis, yet the results vary depending on the assessment tool used [10]. These instruments fall into four models: (1) Phenotype-based models (Fried phenotype, FRAIL, SHARE-FI). (2) Deficit accumulation models (Frailty Index, electronic FI, claim-based scores). (3) Clinical judgment tools, notably the Clinical Frailty Scale (CFS), offer a rapid, pragmatic global assessment. For routine cardiology, the CFS is the most practical choice: it takes <1 min, requires no equipment, and integrates easily into workflow, though it is subjective and should be supplemented by targeted geriatric assessment. (4) Multidimensional geriatric assessment (MPI, CGA). No frailty instrument currently has sufficient evidence to determine whether anticoagulation should be prescribed or withheld on its own. The association between frailty and thromboembolic risk is inconsistent across studies. Differences may partly reflect heterogeneity in frailty definitions, adjustment for conventional stroke risk factors, competing mortality, anticoagulant exposure, and ascertainment of thromboembolic events. Accordingly, frailty should currently be regarded as a modifier of overall prognosis and treatment complexity rather than as a validated independent component of stroke risk prediction [11].

A critical challenge in managing AF in older adults is that frailty assessment tools are not interchangeable. They conceptualize and measure frailty differently, leading to divergent implications for anticoagulation decisions. Phenotypic tools, such as the Fried Frailty Phenotype and the FRAIL scale, focus on physical decline. These have been applied in various settings, including catheter ablation patients and direct oral anticoagulant (DOAC) outpatients [12,13,14,15]. Their primary value lies in identifying patients at risk of functional decline, but their association with bleeding outcomes has been inconsistent. Multidimensional tools adopt a more holistic, deficit accumulation model [16,17,18,19]. The MPI, for instance, integrates cognitive, nutritional, and social domains and has been shown to modify the absolute benefit of DOACs. The Clinical Frailty Scale provides a pragmatic, rapid global assessment of frailty. While highly useful for clinical decision-making, its subjective nature introduces variability. Claim-based indices rely on administrative data. They are powerful for population-level research and have suggested a dose–response relationship with bleeding risk. However, they lack the granularity required for individual point-of-care decisions and may capture disease burden rather than biological frailty per se. The predictive value and clinical utility of these tools vary significantly. For example, the Hospital Frailty Risk Score was found to predict under-treatment in a Danish cohort, while a systematic review showed that frail patients identified by deficit accumulation models, but not phenotypic models, were less likely to receive oral anticoagulation and had worse prognoses [20,21,22,23]. This discrepancy underscores that the choice of a frailty assessment tool is not neutral and must be aligned with the clinical or research objective [24,25]. Clinicians should not treat a patient classified as frail by one tool as equivalent to one classified by another. Instead, the specific frailty construct measured and its proven association with relevant outcomes should guide the decision-making process.

A systematic review indicated that patients assessed as having severe frailty based on the cumulative deficit model had significantly lower oral anticoagulant therapy prescription rates and worse prognoses, whereas no such difference was observed in those assessed by the frailty phenotype. Therefore, frailty should not be used indiscriminately as a gold standard for decision-making [26]. Comprehensive Geriatric Assessment, which identifies and manages modifiable bleeding risk factors, such as polypharmacy, malnutrition, and renal dysfunction, is a core component of anticoagulation decision-making [27]. The AFFIRMO trial, a mobile health integrated management program based on the Atrial Fibrillation Better Care (ABC) pathway combined with Comprehensive Geriatric Assessment, enrolled 1260 patients aged ≥65 years with multimorbid AF (78.7% with hypertension, 40.5% pre-frail, and 13.7% frail). The anticoagulation rate reached as high as 97.3%. This trial is expected to provide evidence-based support for integrated management of elderly multimorbid AF patients in Europe [28]. Table 1 provides a summary of the application scenarios for various frailty assessment tools.

## 3. Decision to Initiate Anticoagulation

### 3.1. Guideline Recommendations

The 2024 ESC/EACTS Guidelines introduce the AF-CARE integrated management framework, recommending the use of the sex-independent CHA2DS2-VA score to guide the initiation of anticoagulation. The guidelines clearly favor DOACs over warfarin, no longer recommend any specific bleeding risk score for decision-making regarding anticoagulation initiation or discontinuation, and emphasize the identification and correction of reversible bleeding risk factors [24].

Evidence suggests that fall risk should not be a barrier to anticoagulation in elderly patients with AF, nor should advancing age per se be regarded as a contraindication. OACs constitute the cornerstone of stroke prevention in all patients with a CHA2DS2-VA score ≥ 2, and DOACs have indicated benefits over warfarin in elderly and frail populations [4,29].

### 3.2. Inadequate Anticoagulant Therapy in Frail Patients

A multicenter Australian study reported that the prescription rate of OACs was significantly lower in frail patients than in non-frail individuals, with an adjusted odds ratio (OR) of 0.58, and frailty was particularly associated with reduced use of DOACs [30]. Another cross-sectional study from Australia similarly suggested a significant negative association between frailty and anticoagulant use, with an adjusted OR of 0.66 [31].

A Vietnamese study suggested that frailty was more prevalent in women, and the OAC prescription rate was significantly lower in women than in men, with the disparity being even greater in the frail subgroup. Each 1-point increase in the Clinical Frailty Scale was associated with a 30% reduction in the odds of receiving OACs in women [32]. A Korean nationwide cohort study indicated that OAC prescription rates were significantly lower in frail patients. However, OAC use significantly attenuated the frailty-associated risks of all-cause mortality, cardiovascular mortality, stroke, and heart failure hospitalization, without significantly increasing major bleeding [22]. A Chinese study of elderly patients reported a frailty prevalence of 50.42% and an anticoagulation rate of only 44.04%. Older age, paroxysmal AF, and a history of bleeding reduced the likelihood of anticoagulation. Anticoagulation reduced thromboembolic events (6.99% vs. 10.98%) but increased bleeding (20.98% vs. 12.72%). Frailty significantly increased the risks of bleeding (HR = 2.294) and all-cause mortality (HR = 4.947) [33].

A Danish nationwide registry study reported that 38.2% of eligible patients did not receive anticoagulation. The overall net benefit of anticoagulation was 0.70%, but this decreased with age > 75 years and increasing frailty [34]. A survey of healthcare professionals showed that DOACs were generally preferred, with apixaban being the most frequently chosen, although the FRAIL-AF trial results have led some clinicians to become more cautious. Moreover, 48.1% explicitly stated that clearer management guidelines for frail elderly patients were needed [35]. A survey of UK physicians revealed that 73% of prescribers had inappropriately reduced the dose at least once, with the main drivers being a history of major bleeding, a history of falls, and severe frailty. Only 32% of physicians fully adhered to guidelines [36].

Inappropriate DOAC dosing was observed in 30% of frail patients [37]. Among DOAC-treated outpatients assessed using the Fried Frailty Phenotype, 41% were classified as frail, of whom 33% received inappropriate doses [13]. A study of nursing home residents revealed that the OAC prescription rate increased from 32.7% to 72.7% over a 16-year period. Paradoxically, greater frailty severity was independently associated with a higher OAC prescription rate (adjusted ORs reaching 4.61–8.42), whereas advanced age and concomitant antiplatelet therapy were barriers to withholding anticoagulation [38]. Approximately 54% of patients with severe frailty, a high comorbidity burden, and severe cognitive and functional impairment were not prescribed OACs, and this non-prescription may reflect clinicians’ reasoned judgment of therapeutic futility or an insufficient net benefit [39]. Consequently, researchers have called for minimizing residual confounding and increasing statistical power in future studies [40,41]. Furthermore, a more refined differentiation of frailty subtypes may facilitate the rational prescribing of anticoagulation [42].

## 4. Selection of Anticoagulant Agent and Dosing

### 4.1. DOAC Versus Warfarin

In patients with AF complicated by frailty, DOACs reduced the risk of stroke or systemic embolism (HR = 0.79), major bleeding (HR = 0.79), intracranial hemorrhage (HR = 0.58), and all-cause death (HR = 0.90) compared with warfarin, without increasing the risk of gastrointestinal bleeding [43,44]. Evidence from large-scale randomized controlled trials and meta-analyses suggests that DOACs have an advantage over warfarin, particularly with an approximately 50% reduction in intracranial hemorrhage risk, although dose adjustment based on renal function is strictly required [29]. However, this benefit may not generalize to the specific subgroup of frail patients already well stabilized on vitamin K antagonist (VKA) therapy. Data from the Italian START registry further indicated that DOACs were associated with significantly lower all-cause and cardiovascular mortality than vitamin K antagonists, without an increased bleeding risk, and the benefit was more pronounced in elderly patients [45].

### 4.2. Comparison of Different DOACs

The ETNA-AF-China study suggests that edoxaban anticoagulation therapy shows favorable overall efficacy and safety [46]. For patients undergoing catheter ablation, the Italian Registry in the setting of AF ablation with rivaroxaban provided real-world evidence on the safety of uninterrupted rivaroxaban in 250 patients [47]. Meta-analyses and observational studies indicate that, among the various DOACs, apixaban has shown lower bleeding rates in real-world studies, particularly for gastrointestinal bleeding [48]. A nationwide Belgian cohort of over 250,000 patients suggests that various DOACs have comparable efficacy, but apixaban was associated with the lowest event rates for major bleeding and gastrointestinal bleeding, suggesting a potential safety advantage [49]. In the cited Medicare analysis, apixaban was the only DOAC demonstrating a statistically significant reduction in the composite endpoint across all frailty strata [50]. A US Medicare analysis suggests that apixaban might reduce the risks of ischemic stroke and major bleeding, whereas the evidence for benefit with dabigatran and rivaroxaban is unclear [20]. Table 2 summarizes the evidence on anticoagulation.

### 4.3. Extremely Advanced Age and Severe Frailty

The ELDERCARE-AF trial suggested that in a specific cohort of older high-risk Japanese patients with AF, ultra-low-dose edoxaban (15 mg once daily) significantly reduced stroke without a significant increase in major bleeding, and there was no significant interaction between the treatment benefit and frailty status [51,52]. However, this very-low-dose regimen should not be extrapolated to routine use in broader populations outside of Japan without consideration of the specific regulatory approvals and differences in baseline patient characteristics.

The EDO-FRAG-001 real-world study (median age 87 years, FRAIL score 3–5) indicated that with appropriate use of edoxaban, the annual incidence of major bleeding was 8.7%, ischemic stroke 1.1%, and all-cause death 18.8%. The leading causes of death were heart failure and infection rather than bleeding [53]. In patients with cognitive impairment, anticoagulation was significantly associated with the composite endpoint of bleeding and death, with a number needed to harm as low as 8.4 [54].

### 4.4. DOAC Dose Reduction

A crucial practical message often overlooked is that frailty itself is not a criterion for DOAC dose reduction. Clinicians frequently and erroneously reduce DOAC doses based on age alone, a history of falls, or a general fear of bleeding. This empiric, non-label-based dose reduction is alarmingly common and leads to inadequate stroke prevention without a proven reduction in major bleeding. The FRAIL-AF trial serves as a powerful reminder that in frail patients stable on VKAs, switching to a DOAC, particularly if not dosed correctly, increased bleeding rather than reducing it, underscoring that frailty should not prompt automatic or empiric dose adjustments. Instead, clinicians must strictly adhere to the label-specific, criteria-based dose reductions and manage modifiable bleeding risks directly. A lower dose should be considered when formal label criteria are met, and the decision must be individualized and shared with the patient.

## 5. Continuation and Monitoring

### 5.1. Risks Versus Benefits

In this context, the net clinical benefit is best quantified by absolute risk differences. For example, the number of ischemic strokes prevented versus the number of major bleeds caused per 100 patient-years of treatment. A Belgian nationwide cohort study indicated that frailty was an independent risk factor for all-cause mortality in patients with AF, but it did not significantly increase the risk of thromboembolism or bleeding [49]. This suggests that the absolute risk reduction from anticoagulation may not be uniformly distributed across frail subgroups. In elderly frail AF patients who continued anticoagulation after discharge, the 1-year all-cause mortality rate was as high as 36.2%, substantially higher than the rates of ischemic stroke (2.9%) and bleeding events (4.7%). Here, the absolute risk of death far outweighed that of the events the therapy aimed to prevent, challenging the net benefit in this specific group. The negative impact of bleeding events on survival (HR = 3.82) was stronger than that of ischemic stroke (HR = 1.82) [55].

The EUROSAF study reported that DOAC use was associated with a 54% lower risk of 1-year all-cause mortality compared with non-users, without an increased bleeding risk, with greater absolute benefits observed in patients with more severe frailty [19]. The TREAT-AF study suggested that although OAC use in frail patients was not significantly associated with a reduced risk of stroke or major bleeding, it was associated with an 18% reduction in 1-year mortality [56].

### 5.2. History of Falls

The ANAFIE registry study suggested that patients with a history of falls within the preceding year had significantly elevated risks of stroke/systemic embolism, major bleeding, cardiovascular death, and all-cause mortality. Notably, incident major bleeding and intracranial hemorrhage frequently occurred in close temporal proximity to new-onset falls or fractures [57]. Compared with warfarin, DOACs were significantly associated with a lower risk of subsequent falls/fractures, potentially attributable to their non-interference with vitamin-K-dependent bone and muscle metabolism.

A cohort study of frail older adults with recurrent falls revealed that OAC use was not independently associated with all-cause mortality. Rather, frailty per se (HR = 74.0) was the predominant predictor of death. In those with concomitant AF, OAC therapy showed a trend toward a reduced mortality risk (HR = 0.52) [58].

A history of falls should therefore trigger assessment and modification of fall-related risk factors rather than automatic withdrawal of anticoagulation. A remote- or single-fall history without major sequelae should prompt evaluation of modifiable risk factors but does not justify withholding anticoagulation, consistent with the ANAFIE registry data.

### 5.3. Post-TAVR Conditions and Heart Failure Hospitalizations

For patients with an independent, ongoing indication for anticoagulation, most commonly atrial fibrillation, OAC should generally be continued post-TAVR unless contraindicated. In this group, the clinical focus is on bleeding risk mitigation rather than OAC withdrawal. Evidence from the OCEAN-TAVI registry, for example, reported a high incidence of late bleeding (7.4% at 3 years) that significantly exceeded that of ischemic stroke and was strongly associated with mortality [59]. Persistent OAC use and frailty were identified as major drivers of bleeding. However, this finding should not be interpreted as a rationale for discontinuing OAC in patients with AF. Rather, it underscores the need for optimized anticoagulant selection, management of modifiable risk factors, and early de-escalation from dual antiplatelet therapy to a single OAC when clinically feasible. The consensus on the management of hospitalized patients with heart failure suggests that in patients with AF and concomitant heart failure, DOACs should be prioritized based on the CHA2DS2-VASc score and that an early rhythm control strategy is superior to rate control alone. Frailty is recommended to be routinely screened using the Fried phenotype or the Clinical Frailty Scale to guide rehabilitation and discharge planning [60].

## 6. The Dilemma of Switching

The FRAIL-AF trial enrolled 1330 frail patients with AF aged ≥75 years and a Groningen frailty indicator (GFI) score of ≥3. Compared with continued VKA therapy, switching to a DOAC significantly increased the risk of major bleeding or clinically relevant non-major bleeding (HR = 1.69, number needed to harm 17), whereas no difference in thromboembolism was observed. However, the trial was prematurely terminated for futility [61]. This trial suggests that frailty denotes a decline in physiological reserve while preserving independence in daily living, whereas disability implies loss of autonomous functional capacity, and the two concepts should not be conflated [62]. In the FRAIL-AF population, the types of DOACs were heterogeneous, with rivaroxaban accounting for half, and no stratified analyses by individual agents were performed to yield reliable conclusions. These results are applicable only to frail patients already stabilized under specialist anticoagulation clinic management and are not generalizable to newly diagnosed patients or those managed in community settings [63]. FRAIL-AF provides evidence against routine switching from well-controlled VKA therapy to a DOAC solely on the basis of age or frailty. The findings should not, however, be extrapolated to anticoagulation-naive patients, patients with poor INR control, or patients with specific indications favoring DOAC therapy.

A nationwide Korean database study suggested that switching from stable warfarin to a DOAC was associated with higher risks of major bleeding (HR = 1.36), thromboembolism (HR = 1.61), and all-cause death (HR = 1.20), with the associations being more pronounced in subgroups of patients aged ≥85 years, females, and those with high frailty scores [64].

Therefore, in frail elderly patients with AF who are stably maintained on VKA with good time in therapeutic range (TTR), routine switching to a DOAC should be avoided. Adjustment should only be considered when there are clear clinical indications [65,66]. Although DOACs remain the preferred anticoagulants for most patients with eligible AF, the 2024 ESC guideline recognizes that continuation of VKA therapy may be considered in clinically stable patients aged ≥75 years with substantial polypharmacy, particularly when anticoagulation control is satisfactory [67]. Table 3 summarizes initiation vs. switching in different conditions.

## 7. Deprescribing and End-of-Life Considerations

### 7.1. Cancer and ACS

Anticoagulation management in cancer-associated AF is exceedingly challenging. Left atrial appendage occlusion (LAAO) may be considered in selected patients with AF who have a contraindication to long-term anticoagulation. Frailty is a critical factor that affects both the perioperative safety and long-term benefit of LAAO and therefore must be weighed carefully when considering this procedure. Key considerations include: (1) procedural risk and life expectancy; (2) the significant risk of competing mortality from cancer or other comorbidities; (3) the need for post-procedure antithrombotic therapy, which may still be required; and (4) the likelihood that the patient will derive a net benefit from stroke prevention within their expected survival horizon [68].

In patients with AF undergoing PCI for ACS, triple antithrombotic therapy should generally be limited to the shortest clinically appropriate duration, usually up to 1 week, with extension to 1 month considered in selected patients at high ischemic/stent-thrombosis risk [69]. In frail patients, the benefit of an invasive strategy should be individually weighed [70]. For patients with AF and NSTE-ACS managed conservatively, triple therapy is not required. Most such patients can be managed with a DOAC alone, with or without a single antiplatelet agent, depending on the timing and urgency of revascularization and the individual thrombotic risk.

### 7.2. Integrated Management of Complex Clinical Manifestations

Evidence from large-scale European registry studies indicates that over 40% of patients with complex clinical profiles present with a history of bleeding, frailty, chronic kidney disease (CKD), or a coexistence of at least two of these features. These characteristics are significantly associated with lower OAC utilization, particularly reduced use of non-VKA DOACs, as well as diminished quality of life and an elevated risk of net clinical outcomes. Notably, patients with ≥2 coexisting features exhibited a more than two-fold increase in risk. Adherence to the ABC pathway was shown to significantly reduce the event rate [71].

### 7.3. Multidrug Therapy

In patients with AF and frailty, the prevalence of multimorbidity and polypharmacy is high, at 86.9% and 81%, respectively [72,73]. Table 4 summarizes the use of anticoagulant therapy in special clinical situations.

## 8. Evidence Limitations

Several limitations warrant consideration. First, frailty definitions are not standardized. Phenotypic, deficit accumulation, and clinical judgment tools capture different constructs, complicating cross-study comparisons and clinical translation. Second, observational biases are pervasive. The frailest patients are routinely excluded from RCTs, those denied anticoagulation often have worse perceived prognosis, and comparisons of treatments are susceptible to immortal time and dose selection biases, the latter manifesting as frequent inappropriate DOAC dose reductions in frail individuals. Third, competing mortality from non-cardiovascular causes frequently outweighs stroke risk in severe frailty, yet many analyses do not formally account for this. Fourth, the ELDERCARE-AF trial, pivotal for very-low-dose edoxaban, was conducted exclusively in Japan and may not generalize to other regions. Fifth, short follow-up in many observational studies limits assessment of long-term outcomes and dynamic frailty transitions. Collectively, these limitations underscore that current evidence must be interpreted cautiously and reinforce the need for individualized, patient-centered decision-making. Future prospective studies with standardized frailty metrics and adequate representation of severely frail patients are needed.

## 9. Discussion

This review synthesizes the current evidence on anticoagulation in the clinically challenging population of older patients with AF and frailty. Several key themes emerge that have implications for clinical practice and future research.

An area of agreement is the advantage of DOACs over warfarin in this population. Meta-analyses and large observational studies suggest that DOACs are associated with a significant reduction in the risk of stroke, intracranial hemorrhage, and all-cause mortality. Among the DOACs, some observational and real-world data suggest that apixaban may be associated with a more favorable safety profile, particularly regarding major and gastrointestinal bleeding [74]. This relative benefit is supported by multiple real-world cohorts and is a key factor in clinical decision-making. Furthermore, for the subset of the oldest and most severely frail patients who are ineligible for standard-dose anticoagulation, the ELDERCARE-AF trial provides a clear evidence-based option specific to the Japanese context: very-low-dose edoxaban. Its application outside Japan requires careful consideration of regulatory status and clinical appropriateness, as it should not be assumed as a universally applicable routine strategy. When the acquisition costs of anticoagulants are excluded, apixaban incurs the lowest non-drug costs [75].

Another layer of complexity stems from the finding that the net clinical benefit defined as the absolute difference between prevented ischemic strokes and caused major bleeds diminishes as frailty severity increases. In patients with the most severe frailty, the benefit may become marginal or even vanish, as non-cardiovascular deaths become the predominant competing risk. This highlights that while frailty should not preclude anticoagulation, its severity is a crucial modifier of the risk/benefit calculus. The frailty paradox, where greater frailty can be associated with a higher absolute benefit in some studies but also higher bleeding risk, is likely a result of the heterogeneity within the frail population and underscores the need for multidimensional assessment. Anticoagulation decisions should integrate life expectancy, functional status, and patient preferences [76]. Whether frailty independently affects drug metabolism remains unclear. Tools such as the FORTA list emphasize medication appropriateness rather than defining polypharmacy solely by pill count [77].

Despite these areas of consensus, the evidence is not monolithic, and the most significant point of contention arises from the FRAIL-AF trial. This high-quality RCT directly challenges the prevailing preference for DOACs by demonstrating that switching from a stable VKA regimen to a DOAC in a frail population increased, rather than decreased, bleeding risk [78]. This finding must be interpreted cautiously and contextualized. It is crucial to emphasize that FRAIL-AF enrolled a specific subset of patients: older, frail, and already clinically stable on INR-guided VKA therapy. The trial’s message is not that DOACs are inferior to VKAs in all frail patients but that routine switching from well-controlled VKA therapy should be avoided. Extrapolating these results to anticoagulation-naive patients, those with poor INR control, or those with specific clinical indications favoring DOACs is not justified. The results therefore do not refute the benefit of initiating DOACs in anticoagulant-naive frail patients, but they strongly argue against a policy of routine, automatic switching for stable patients already achieving good effect. This critical distinction is increasingly recognized in updated guidelines, which now caution against indiscriminate switching. The 2024 ESC Class IIb recommendation reflects this nuanced interpretation, and our review further supports this individualized approach for patients stable on VKA therapy. Clinical decision-making should transition from a guideline-driven approach to one driven by patient values [79]. This review underscores significant limitations in the existing evidence base, which must be acknowledged. First, the vast majority of data are derived from observational, retrospective, or post-hoc analyses. These studies are inherently susceptible to selection bias and residual confounding, limiting their ability to establish causality. Second, there is a striking heterogeneity in the definitions and tools used to assess frailty, ranging from simple phenotypic scales to complex deficit accumulation indices. This lack of standardization makes it difficult to compare results across studies and to translate findings into a unified clinical algorithm. The predictive value of a given tool may vary significantly depending on whether it models frailty as a phenotype of physical decline or as a cumulative burden of health deficits. Therefore, the choice of assessment tool is not neutral and can influence both the estimation of prognosis and the decision to treat.

The practical implication of this evidence is a paradigm shift from a guideline-driven approach to a patient-value-driven approach. For the clinician, this means moving beyond the CHA_2_DS_2_-VA and HAS-BLED scores as primary decision makers. Frailty should complement, rather than replace, validated thromboembolic risk assessment. Instead, a dynamic and personalized strategy is required. This begins with a formal frailty assessment using a validated clinically feasible tool like the Clinical Frailty Scale, conducted upon diagnosis and reassessed dynamically. This assessment must be integrated into shared decision-making, where the patient’s life expectancy, functional status, personal goals, and values are given as much weight as the clinical risk scores. It is therefore recommended that future studies adopt multicenter protocols, longitudinal follow-up, systematic reviews, or additional randomized controlled trials to further validate the results [80]. Current randomized controlled trials provide markedly insufficient evidence for elderly, frail, and multimorbid patients. Accordingly, more geriatric-oriented prospective studies, standardized frailty assessment frameworks, and real-world evidence are urgently needed to fill this knowledge gap [81].

The key question for clinicians is not simply which drug but how to integrate frailty into a practical, patient-centered decision-making framework. To achieve the objective stated in our title and translate the evidence into practice, we propose a three-step clinical thinking framework to help navigate these complex decisions. This framework is intended as a pragmatic guide to structure clinical reasoning, rather than as a set of validated therapeutic thresholds. We suggest using a clinically feasible tool such as the Clinical Frailty Scale to categorize patients into descriptive tiers. This stratification can serve as a common language for the clinical team. For heuristic purposes, patients may be considered as: pre-frail to mildly frail (1–4), where standard anticoagulation is generally appropriate after weighing stroke and bleeding risks; moderately frail (5–6), where the balance of competing risks is less certain and a more cautious reassessment is warranted; and severely frail (≥7), where the net clinical benefit is highly uncertain due to competing mortality, and the focus should shift toward careful consideration of patient values, life expectancy, and functional goals. Crucially, these categories are descriptive and are not validated cutoffs to mandate or withhold therapy. Regarding agent choice, DOACs are generally recommended, with some observational data suggesting a favorable bleeding profile for apixaban, although this advantage should be interpreted cautiously given the lack of dedicated randomized trials in frail populations. Very-low-dose edoxaban (15 mg once daily) may be considered for a highly selected subset of severely frail or high-bleeding-risk patients, but this strategy should be restricted to the specific population and regulatory context of the ELDERCARE-AF trial and should not be extrapolated broadly to all frail individuals. However, stable warfarin users with good TTR should not be routinely switched to DOACs per FRAIL-AF. Additionally, comorbidities modify decisions: fall risk alone should not preclude anticoagulation; heart failure, polypharmacy, and drug interactions require proactive management within a holistic ABC care pathway. Ultimately, frailty is not a binary on/off switch but a dynamic modifier, and decisions must be collaborative, individualized, and regularly revisited.

## 10. Conclusions

Managing anticoagulation in older patients with atrial fibrillation and frailty requires an individualized approach that moves beyond simplistic risk scores.

Most patients with AF and frailty derive a net clinical benefit from anticoagulation, but this benefit must be weighed against the severity of frailty. The available evidence, while largely observational, supports the use of DOACs over warfarin, with apixaban showing favorable safety signals. However, based on the FRAIL-AF trial, routine switching from well-controlled warfarin therapy to a DOAC solely on the basis of age or frailty is not recommended, as this increases bleeding risk. For the most severely frail or high-bleeding-risk patients who are ineligible for standard doses, consider very-low-dose edoxaban, acknowledging that this recommendation is primarily based on the ELDERCARE-AF trial conducted in a Japanese population and should be individualized within its appropriate clinical and regulatory framework. Clinicians should perform a systematic frailty assessment, apply the evidence-based rules above to select the initial therapy or decide against switching, and use shared decision-making to align the plan with the patient’s values, life expectancy, and functional goals. This patient-value-driven framework is a strategy to maximize benefit and minimize harm in this complex and vulnerable population.

## Figures and Tables

**Table 1:** Frailty assessment tools and their utility in anticoagulation decision-making for atrial fibrillation.

Tool	Model Basis	Assessment Content/Time	Association with Anticoagulation Outcomes	Applications/Pros & Cons
Fried phenotype/5-item frailty screening	Phenotypic (weight loss, grip strength, gait speed, activity, exhaustion)	Requires measurements; 5–10 min	Predicts bleeding (OR ≈ 2.5) and death; weak for thromboembolism; high negative predictive value	Suitable for outpatient/inpatient; needs equipment; used in ablation patients
Clinical Frailty Scale	Clinical judgment (1–9 scale)	Rapid, <1 min	Severe frailty (≥7) linked to lower OAC prescribing and higher mortality; guides switching decisions	Simple, pragmatic for emergency/ward; somewhat subjective
FRAIL scale	Phenotypic (5 self-reported items)	Self-reported; 2 min	HAS-BLED-F (combining FRAIL) showed 90.5% sensitivity for bleeding prediction	Suitable for community/outpatient; no equipment; validated in Chinese elderly
SHARE-FI	Phenotypic (age, grip, weight, gait, etc.)	Requires measurements	In ≥80-year-old edoxaban users, frailty was not significantly associated with composite outcomes	Validated in European older population; useful for very old
Multidimensional prognostic index	Deficit accumulation (cognition, nutrition, comorbidity, social support, etc.)	Comprehensive; 10–15 min	In EUROSAF, DOAC absolute benefit increased with frailty severity	Comprehensive, ideal for inpatient geriatric assessment; time-consuming
Electronic Frailty Index/claims-based	Deficit accumulation (ICD codes, prescriptions, etc.)	Automated, using databases	Dose–response relationship with bleeding (severe: HR = 1.86); weak association with stroke	Suitable for large database research; not for bedside use
Hospital Frailty Risk Score	ICD-code-based cumulative model	Automated	In Danish cohort, net benefit of anticoagulation lost at ≥15 points; Korean data linked to under-treatment	Suitable for retrospective database studies; not for individual point-of-care

DOAC, direct oral anticoagulant; OR, odds ratio; HR, hazard ratio. Different tools capture different frailty dimensions and are not interchangeable.

**Table 2:** Key clinical evidence on anticoagulation in frail patients with atrial fibrillation.

Study/Registry	Design	Population/Frailty Assessment	Anticoagulation Comparison	Key Frailty-Related Outcomes
FRAIL-AF	RCT	≥75 years, GFI ≥ 3, stable on VKA	Switch to DOAC vs. continue VKA	In patients stable on VKA, switching to DOAC increased major/CRNM bleeding; supports avoiding routine switching in well-controlled VKA users
ELDERCARE-AF	RCT	≥80 years, high bleeding risk, ineligible for standard doses	Very-low-dose edoxaban 15 mg vs. placebo (Japanese population)	Significant reduction in stroke/embolism (frail: 7.1% → 2.5%/year); no significant increase in major bleeding
EUROSAF	Prospective	Hospitalized older AF, MPI-stratified	DOAC vs. no anticoagulation	DOAC reduced 1-year mortality by 54% (HR = 0.46); absolute benefit greater with more severe frailty (NNT = 4)
Belgian nationwide cohort	Retrospective	>250,000 AF patients, frailty defined by claims	Individual DOACs vs. VKA	Frailty itself did not increase thromboembolism or bleeding; DOACs superior to VKA, apixaban lowest bleeding risk
Korean nationwide	Retrospective	≥75 years, switched from stable warfarin	Switch to DOAC vs. maintain warfarin	Switching increased major bleeding (HR = 1.36), thromboembolism (HR = 1.61), and death (HR = 1.20); more pronounced in ≥85 years, females, high frailty scores
ETNA-AF-China	Prospective	Chinese AF patients, 6.3% with perceived frailty	Edoxaban	Frailty independently predicted all-cause death (HR = 2.14) and cardiovascular death (HR = 2.94); overall event rates low
US Medicare sequential analysis	Sequential simulated trial	Older AF, claim-based Frailty Index	Apixaban, dabigatran, rivaroxaban vs. warfarin	Apixaban reduced stroke (HR = 0.73) and bleeding (HR = 0.63) in frail; benefit for other DOACs unclear

GFI, Groningen frailty indicator; MPI, multidimensional prognostic index; VKA, vitamin K antagonist; DOAC, direct oral anticoagulant; CRNM, clinically relevant non-major; RCT, randomized controlled trial; AF, atrial fibrillation; HR, hazard ratio; NNT, number needed to treat.

**Table 3:** Clinical decision framework for anticoagulation in atrial fibrillation and frailty.

Clinical Scenario	Decision Type	Evidence-Based Suggestion
Anticoagulation-naive frail patient	Initiation	Initiate OAC when thromboembolic risk warrants it (CHA_2_DS_2_-VA ≥ 2). Frailty alone should not preclude treatment. Prefer DOACs over warfarin based on superior efficacy/safety.
Stable VKA with excellent TTR	Switching	Avoid routine switching to DOAC. FRAIL-AF showed increased bleeding without thromboembolic benefit. Continue VKA.
Poorly controlled VKA	Switching	Switching to DOAC may be advantageous if VKA control cannot be improved, weighing potential benefit against the risks observed in FRAIL-AF.
Prior intracranial haemorrhage/high ICH risk	Initiation or Switching	DOACs are preferred due to lower ICH risk. This applies to both initial therapy and switching from VKA.
Severe renal dysfunction	Initiation or Switching	Choice becomes substantially more complex. Dose adjustments are mandatory; consider pharmacokinetics. Multidisciplinary input advised.
Limited life expectancy/severe frailty	Initiation or Switching	Net clinical benefit may be uncertain or vanish. Focus on shared decision-making, incorporating patient values, functional status, and life expectancy. Deprescribing may be appropriate.

DOAC, direct oral anticoagulant; ICH, intracranial haemorrhage; OAC, oral anticoagulant; TTR, time in therapeutic range; VKA, vitamin K antagonist.

**Table 4:** Anticoagulation strategies in special clinical scenarios with frailty.

Clinical Scenario	Key Anticoagulation Recommendations	Important Evidence/Cautions
≥85 years with severe frailty	Standard label DOAC dosing should be used unless specific dose reduction criteria are fulfilled; ELDERCARE-AF provides evidence for edoxaban 15 mg once daily only in a highly selected Japanese population ≥ 80 years considered unsuitable for standard-dose anticoagulation; these findings should not be extrapolated routinely to other populations	ELDERCARE-AF supports very-low-dose (Japanese cohort); EDO-FRAG-001 showed main causes of death were heart failure/infection, not bleeding
History of falls/high fall risk	Fall risk alone should not preclude anticoagulation; DOACs associated with lower fall/fracture risk than warfarin	ANAFIE registry showed DOACs linked to fewer subsequent falls/fractures; but new falls may trigger bleeding events
Post-TAVR	Continued OAC is major driver of late bleeding; frailty independently increases late bleeding (OR = 1.55) and mortality (HR = 5.40)	Balance anticoagulation against bleeding risk in older, frail patients; de-escalation strategies may be considered
Heart failure hospitalization	Initiate DOAC based on CHA_2_DS_2_-VA; early rhythm control preferred over rate control; routine frailty screening (Fried/CFS) guides rehabilitation and discharge planning	Consensus recommendation; dynamic frailty changes affect discharge planning and readmission risk
Cancer-associated AF	Long-term anticoagulation challenging; LAAO may be considered only in selected patients with a clear contraindication to long-term OAC	Strict selection (life expectancy > 1 year, acceptable functional status); multidisciplinary timing (chemotherapy pause/remission)
NSTE-ACS with AF	Avoid potent P2Y_12_ inhibitors; triple therapy ≤ 1 month, then DOAC + clopidogrel up to 12 months; invasive strategy individualized in frail patients	Benefit–risk trade-off; Comprehensive Geriatric Assessment may guide revascularization timing
Multimorbidity and polypharmacy	Monitor P-gp/CYP3A4 interactions; consider drug concentration monitoring if needed	Adjusting administration time helped anticoagulation; FORTA list emphasizes medication appropriateness, not just pill count

AF, atrial fibrillation; DOAC, direct oral anticoagulant; LAAO, left atrial appendage occlusion.

## Data Availability

All data generated or analyzed during this study are included in this published article.

## Abbreviations

The following abbreviations are used in this manuscript:

AF	atrial fibrillation
MPI	multidimensional prognostic index
HR	hazard ratio
DOAC	direct oral anticoagulant
VKA	vitamin K antagonist
GFI	Groningen frailty indicator
TTR	time in therapeutic range
LAAO	left atrial appendage occlusion
CKD	chronic kidney disease
ABC	Atrial Fibrillation Better Care
OR	odds ratio
